# Molecular and biochemical basis of softening in tomato

**DOI:** 10.1186/s43897-022-00026-z

**Published:** 2022-02-11

**Authors:** Duoduo Wang, Graham B. Seymour

**Affiliations:** 1 College of Chemistry and Life Sciences, Zhejiang Normal University, Jinhua, Zhejiang, 321004 China; 2 School of Life Sciences, Jiangsu University, Zhenjiang, 212013 China; 3 Divison of Plant and Crop Sciences, University of Nottingham, Sutton Bonington, Loughborough, Leics, LE12 5RD UK

**Keywords:** Tomato, Cell walls, Softening, Pectin, Ripening

## Abstract

We review the latest information related to the control of fruit softening in tomato and where relevant compare the events with texture changes in other fleshy fruits. Development of an acceptable texture is essential for consumer acceptance, but also determines the postharvest life of fruits. The complex modern supply chain demands effective control of shelf life in tomato without compromising colour and flavour.

The control of softening and ripening in tomato (*Solanum lycopersicum*) are discussed with respect to hormonal cues, epigenetic regulation and transcriptional modulation of cell wall structure-related genes. In the last section we focus on the biochemical changes closely linked with softening in tomato including key aspects of cell wall disassembly. Some important elements of the softening process have been identified, but our understanding of the mechanistic basis of the process in tomato and other fruits remains incomplete, especially the precise relationship between changes in cell wall structure and alterations in fruit texture.

## Introduction

Controlling shelf life in cultivated tomato (*Solanum lycopersicum*) and other fruits is essential to the successful function of the modern fresh market supply chain. Substantial losses during transportation, susceptibility to post harvest diseases and limited keeping quality, result from fruits that ripen and soften rapidly. This has led to the use of a range of natural ripening mutations in commercial tomato breeding that slow the ripening and softening processes. The best known of these is the *ripening inhibitor* (*rin*) mutation. The use of the *rin* mutation in commercial practice has been very important in extending tomato shelf life, but it has detrimental effects on colour and flavour (Kitagawa et al., 2005). The goal for breeders is to control specifically the softening process in tomato, while maintaining other aspects of normal ripening. A similar strategy is needed to extend postharvest life in other fleshy fruits.

In this review, we draw together the latest evidence to explain the molecular and biochemical mechanisms involved in the softening process in tomato and where relevant ask if the events occurring in tomato can help us understand the softening process in other fruit species. The review is structured so that the control of softening in tomato is discussed in relation to a molecular framework describing ripening. We review first hormonal, epigenetic and transcriptional control and then the expression of genes modifying cell wall structure and the links between these events and softening. Readers unfamiliar with plant cell wall structure and wall modifying enzymes in tomato may also want to refer to Anderson and Kieber (2020) and Tomato Genome Consortium (2012, supplemental text).

## Molecular mechanisms controlling ripening and softening

Fruit softening in tomato and other fleshy fruit species is brought about by processes that include cell wall disassembly as a result of the action of cell wall enzymes and other cell wall factors and alterations in fruit cuticle structure. Modifications that affect the cuticle have been the subject of several excellent reviews (Lara et al., 2019; Martin & Rose, 2014) and will not be discussed further here. Cell wall disassembly in tomato has been studied extensively over many decades. The cell walls of the tomato fruit, including those of the pericarp and columella, generally lack lignin and are rich in pectin, xyloglucan and cellulose, with a smaller percentage of structural and other proteins. More than 50 cell wall structure-related genes are known to be expressed in developing and ripening tomato fruits and these include enzymes that act on all the main polysaccharide domains within the cell wall and wall modifying proteins such as expansins (Tomato Genome Consortium, 2012).

**Fig. 1:**
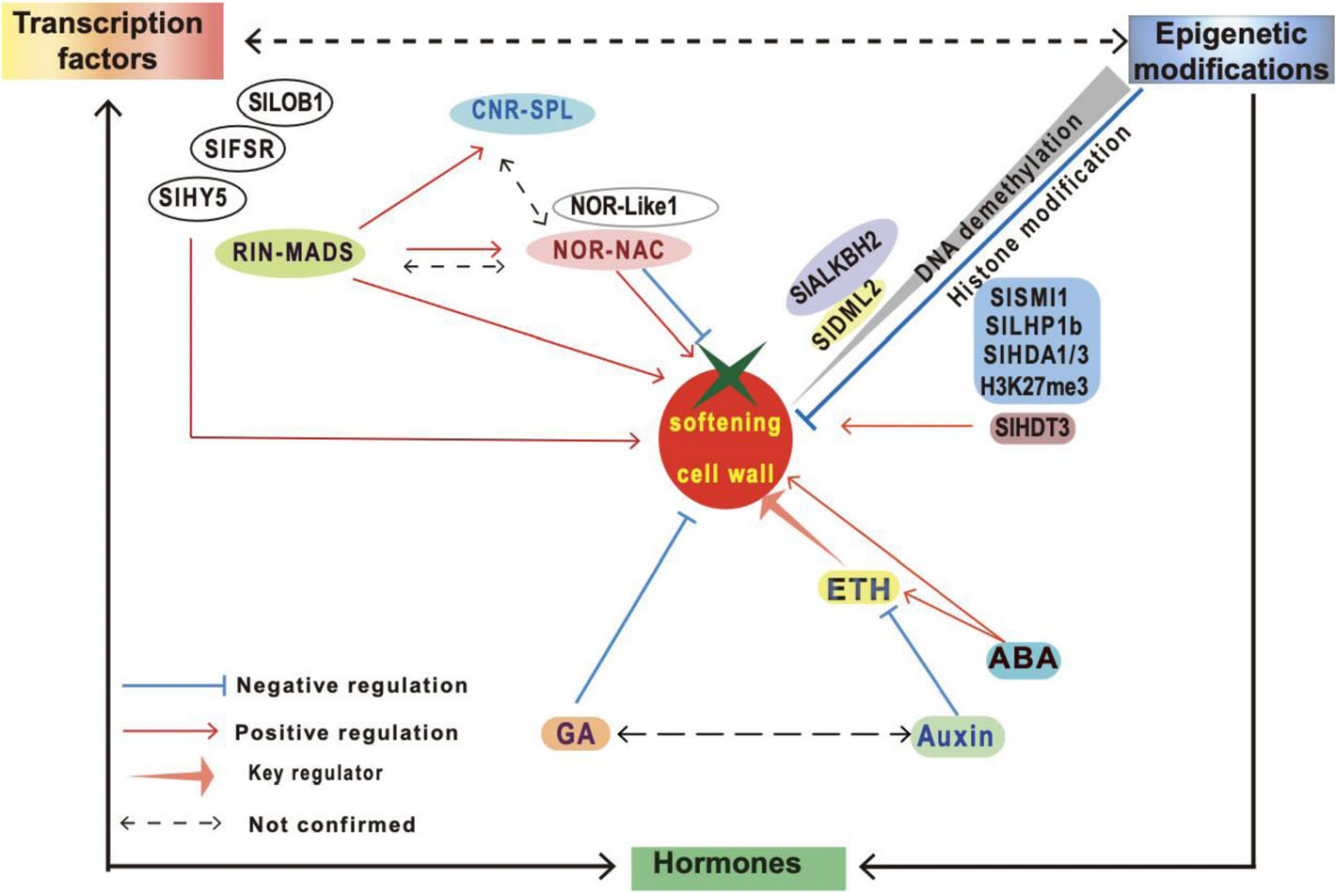
Hormone, transcription and epigenome changes regulating softening in tomato. Tomato fruit softening is directly or indirectly regulated by a range of transcription factors (TFs). These include RIN-MADS, SPL-CNR and TFs from the NAC-box family such as NOR-Like1 along with SlLOB1, SlFSR and SlHY5. Ripening-associated softening processes are initiated and modulated by complicated network of feedback and crosstalk among different phytohormones including ethylene (ETH), abscisic acid (ABA), auxin and gibberellic acid (GA). Direct links between hormones and fruit texture regulation have been observed during tomato fruit ripening. Modifications to the epigenome especially DNA methylation and histones and RNA methylation are associated with ripening-related softening. SlDML2 is a key player regulating demethylation during tomato ripening. SlALKBH2, active m6A RNA demethylase, is necessary for normal tomato fruit ripening by direct targeting and stabilising transcript of *SlDML2.* Histone deacetylases SlHDA3 and SlHDA1, polycomb-group proteins SlMSI1 and SlLHP1b along with epigenetic mark H3K27me3 function as negative regulators of fruit softening, while histone deacetylases SlHDT3 plays a positive role in tomato fruit softening

There is strong evidence that cell wall disassembly is responsible for a major portion of fruit softening and that this is initiated by a complex interplay between hormonal cues, changes in the epigenome and tightly regulated expression of numerous transcription factors (TFs) and downstream genes (Fig. 1).

### Hormonal cues

Plant hormones, such as ethylene, act as endogenous and exogenous cues that govern the transition stage from fruit development to ripening. Extensive studies have shown that fruit ripening is modulated by a complicated network of feedback and crosstalk among different phytohormones including ethylene, abscisic acid (ABA) and auxin (McAtee et al., 2013; Bai et al., 2021) (Fig. 1). Indeed, the control of ripening in climacteric fruit, by exposure to applied ethylene, is a well-established commercial practice, especially for initiating and coordinating the ripening of fruits such as bananas. Ethylene is also used to induce ripening in tomato in commercial practice in some countries.

Ethylene is a major cue triggering ripening of fruits that show a respiratory climacteric, such as tomato (Fenn & Giovannoni, 2021). However, in tomato and other climacteric fruits, there is substantial evidence for the involvement of additional hormone signalling pathways alongside ethylene. For example, ABA may function upstream of the ethylene pathway to control ripening (Zhang et al., 2009a, 2009b; Mou et al., 2016). Links between ABA and the regulation of tomato texture have been observed (Zaharah et al., 2013; Sun et al., 2012; Ji et al., 2014). Silencing of the tomato *9-CIS-EPOXYCAROTENOID DIOXYGENASE* (*SlNCED1*) and *ABA 8′-HYDROXYLASE* (*SlCYP707A2*) genes, which are genes involved in ABA biosynthesis and catabolism, resulted in elevated pectin levels and improved texture and shelf life and several genes encoding major cell wall catabolic enzymes were down regulated (Sun et al. 2012; Ji et al., 2014). Manipulating *PYROBACTIN RESISTANCE-LIKE (SlPYL9),* a gene which encodes an ABA receptor, has revealed a role for SlPYL9 in regulating ABA signalling and ripening in tomato by affecting expression of ripening-related genes involved in ethylene production and cell wall modification (Kai et al., 2019).

Observations linking ABA with tomato softening indicate genes controlling fruit texture are ABA responsive. This is consistent with function characterisation of the ripening-related transcription factor SlNAC1 in transgenic tomato overexpression and silencing lines. Experiments showed the softening rate was inconsistent with the rate of ethylene production and carotenoid accumulation, but consistent with ABA levels, supporting a role for ABA-related softening pathways in tomato (Ma et al., 2014; Meng et al., 2016). Expression of NAC genes has also been linked with ethylene and ABA (Kou et al., 2021). Re-examining the role of *SlNAC084*, *SNAC4* and *SNAC9* in regulating tomato fruit ripening, using gene silencing technologies, demonstrated that NAC TFs could function as positive regulators of tomato ripening by regulating ethylene synthesis (Zhu et al., 2014; Kou et al., 2016). NAC TFs were also reported to regulate softening through an ABA-dependent pathway. Silencing SNAC4 inhibit tomato fruit softening through down regulating the level of ABA, while silencing SNAC9 accelerate softening by inducing the accumulation of ABA (Kou et al., 2018). Kou reported that these NAC TFs regulate softening through the modulation of cell wall modifying enzymes including polygalacturonase (PG), and pectate lyase (PL) that act to depolymerise pectic polysaccharides (Kou et al., 2018). The relative expression levels of these genes were downregulated in SNAC4-silenced lines and upregulated in SNAC9-silenced lines.

In fruits other than tomato, there is also evidence for an important role for ABA in softening. For example, in strawberry, a non-climacteric fruit, a C-type MADS-box gene, *FaSHP* controlled the expression of cell wall structure-related genes either directly or indirectly through interaction with hormones including auxin and ABA (Daminato et al., 2013). In addition, during strawberry fruit ripening, several genes encoding cell wall modifying genes such as *RHAMNOGALACTURONATE LYASE* (Fa*RGLYASE1*), β-*GALACTOSIDASE* (*FaβGAL4*) are positively regulated by ABA and negatively regulated by auxin (Molina-Hidalgo et al., 2013; Paniagua et al., 2016). ABA is associated with fruit ripening in watermelon and a good correlation was observed between the expression patterns of key genes involved in ABA metabolism and signal transduction and genes likely involved in softening. These included genes encoding a putative β-galactosidase, glucan synthase, a pectinacetylesterase-like protein, an endoglucanase and a mannosyl-oligosaccharide 1,2- α-mannosidase (Wang et al., 2017). A more recent study of the synergistic role of sucrose and ABA in the regulation of strawberry fruit ripening indicated that cell-wall metabolism-related genes including *V-MYB AVIAN MYELOBLASTOSIS VIRAL ONCOGENE HOMOLOGUE* (*MYB5*), *CELLULASE 1* (*CEL1*), and *CELLULASE 2* (*CEL2*) were significantly up-regulated by ABA treatments, thus accelerating ripening in strawberry (Luo et al., 2020).

Auxin can act to delay ripening in tomato and other fruits (McAtee et al., 2013; Kumar et al., 2014; Castro et al., 2021). The action of auxin appears to involve repressing the expression of genes responsible for regulating ethylene synthesis, cell wall degradation and even DNA demethylation (Li et al., 2016). Auxin counteracts the effects of ethylene to prolong shelf life in papaya by repressing the transcription of ripening-related genes including those encoding cell wall enzymes via the downstream Auxin Response Factors-Ethylene Insensitive3-Like (CpARF2-CpEIL1) transcriptional complex (Zhang et al., 2020).

Gibberellins (GA) also participate in modulating tomato fruit ripening via an interplay with ethylene. In several recent studies GA has been demonstrated to play a negative role in regulating tomato ripening. It is associated with suppression of the expression of ripening associated transcription factors and inhibiting ethylene production (Li et al., 2019). More recently, a major quantitative trait locus (QTL), termed qFIRM SKIN 1 (qFIS1) determining fruit firmness in tomato, was identified, and a key gene (*FIS1*) within the QTL was cloned. This *FIS1* gene encodes a GA2-oxidase, an enzyme that inactivates endogenous GAs and their precursors (Li et al., 2020a). Knockout of the *FIS1* gene enhanced fruit firmness by influencing cuticle accumulation and deposition, while maintaining other aspects of ripening, proving a direct role of GA regulating fruit firmness and providing new insights for targeted control of tomato fruit firmness (Li et al., 2020a).

Further details of the interaction between plant hormones and the molecular and biochemical events involved in fruit softening remain to be elucidated in tomato and other fleshy fruits. Modulation of hormone biosynthesis and signalling represents an important source of potential control points for fine tuning texture changes and shelf life.

### Epigenome priming for ripening and softening

Modifications to the epigenome can have a profound effect on gene expression. Changes to the epigenome can include events such as DNA methylation, chromatin remodelling, histone modifications, the production of non-coding RNAs (ncRNAs), and RNA methylation. Many of these events have now been shown to be associated with the progression of normal ripening and softening in tomato and other fruits (Giovannoni et al., 2017).

A reduction in DNA methylation in the regulatory regions of ripening-related genes in tomato has been reported by several labs (Hadfield et al., 1993; Manning et al., 2006) including a comprehensive and detailed study by (Zhong et al., 2013). A key player in regulating DNA methylation in ripening tomato is the DEMETER-like DNA demethylase (DML) SlDML2. Down regulation of *SlDML2* by RNAi led to ripening defects that were correlated with hypermethylation of promoters and repression of genes necessary for fruit ripening and softening (Liu et al., 2015). Interestingly, reduced DNA methylation is also associated with ripening-induced repression of many genes, such as those involved in photosynthesis and cell wall synthesis and organisation (Lang et al., 2017).

In contrast to a global loss of DNA methylation detected during tomato ripening, DNA methylation undergoes a genome-wide increase during ripening in non-climacteric fruit such as sweet orange (Huang et al., 2019). The data of Huang suggests that the ripening-induced DNA hypermethylation potentially contributes to sweet orange fruit softening through repression of genes involved in maintaining cell wall organisation during development (Huang et al., 2019).

RNA may also show changes in methylation and these have been associated with ripening control. For example, m6A mRNA methylation has been shown to exhibit dynamic changes during tomato ripening and can regulate the ripening processes via the interplay with DNA methylation (Zhou et al., 2019). Modulation of an active m6A RNA demethylase, SlALKBH2, in tomato fruit using CRISPR/Cas9 indicated that SlALKBH2-mediated m6A demethylation is necessary for normal ripening. SlALKBH2 has the ability to bind the transcript of *SlDML2* and one mechanism is the direct targeting and likely stabilising of the transcript of *SlDML2* (Zhou et al., 2019; Lang et al., 2017).

Histone post-translational modifications and the remodelling of chromatin structure seem also to impact fruit softening. Histone acetylation is generally associated with increased gene activity. Several genes encoding histone deacetylases were discovered to regulate tomato ripening, although genes from different subfamilies might play contrasting roles. For example, histone deacetylases SlHDA3 and SlHDA1 from RPD3/HDA1 subfamily functioned as negative regulators of fruit softening by repressing genes involved in cell wall metabolism. However, SlHDT3 from the HD2 (Histone Deacetylase2) subfamily played a positive role in regulating fruit softening through the activation of the same set of cell wall related genes regulated by SlHDA1/3 (Guo et al., 2017a, 2017b, 2018). These observations indicate that multiple epigenetic markers may act in a cooperative way in regulating ripening and softening-related gene expression (Fig. 1).

The fruit ENCODE project (Lü et al., 2018) revealed interesting epigenetic marks associated with ripening and softening in a range of fruits. The project involved generating a comprehensive annotation of functional elements in seven climacteric fruit species by constructing a multidimensional dataset encompassing 361 transcriptome, 71 accessible chromatin, 147 histone modification and 45 methylome profiles. It revealed that another histone modification marker, H3K27me3, that is associated with gene silencing, also plays a conserved role in restricting the expression of ripening genes and their orthologues in tomato a fleshy ethylene-dependent fruit and in ethylene-independent and dry fruits (Lü et al., 2018).

Furthermore, polycomb-group (PcG) proteins within the polycomb repressive complex act as repressors of gene expression via histone modifications (Mozgova & Hennig, 2015). Several recent studies have provided new insights into the PcG-mediated epigenetic regulation of climacteric fruit ripening. Transgenic studies indicated that PcG protein SlMSI1 acted upstream of RIN and negatively regulated fruit ripening by repressing *RIN* and its downstream targets including genes encoding cell-wall modifying factors (Liu et al., 2016). More recently, a tomato Polycomb Repressive Complex 1 (PRC1)-like protein, Heterochromatin Protein 1b (SlLHP1b) was shown to repress fruit ripening through colocalization with the epigenetic mark H3K27me3 (Liang et al., 2020).

### Transcriptional control of fruit softening

A range of ripening related TFs have been reported to be involved in regulating tomato fruit softening. The links between TFs and softening were initially made through investigations on spontaneous mutations in tomato where the fruit failed to ripen normally and remained firm. The most thoroughly characterised mutations include *ripening inhibitor* (rin), *non-ripening* (*nor*) and *Colourless non-ripening* (*Cnr*) (Vrebalov et al., 2002; Manning et al., 2006). The genes underlying these mutant loci have been cloned and it is now apparent that all these mutations are likely gain of function changes (Wang et al., 2020; Li et al., 2021). The *RIN*, *NOR* and *CNR* genes are all ripening-related TFs. They all have important roles in the ripening process (Fujisawa et al., 2011, 2013), but they are among many TFs that are expressed during tomato ripening. The tomato genome project and associated transcriptomic studies has revealed there are several hundred TFs involved in the ripening and at least 50 cell wall structure-related genes (Tomato Genome Consortium, 2012) demonstrating the complexity of this developmental process.

In a recent study (Lü et al., 2018) three general types of transcriptional feedback circuits controlling ethylene-dependent fruit ripening have been systematically characterised using an ENCODE-style functional genomic approach. Examples are the tomato MADS-type circuit, peach NAC-type circuit and banana dual-loop circuit. Climacteric fruits which have undergone a recent whole genome-duplication (WGD) such as tomato utilise the MADS transcription factor RIN to form a positive feedback loop to generate autocatalytic ethylene to regulate ripening by activating downstream genes (Lü et al., 2018). Genomes of climacteric fruit species that have not undergone a recent WGD seem to utilise a NAC TFs instead of MADS TFs in order to generate a positive feedback circuit with ethylene to regulate ripening. Banana, a monocot, which has experienced recent WGDs, operates a dual-loop system involving both the NAC and MADS genes.

Analysis has demonstrated that the RIN TF interacts directly with the regulatory regions of genes involved in cell wall remodelling such as *PG, β-GALACTOSIDASE 4 (TBG4), ENDO-(1,4)-Β-MANNANASE 4 (LeMAN4)* and *α-EXPANSIN 1 (LeEXP1), CEL2, XYLAN 1,4-BETA-XYLOSIDASE1 (XYL1)* (Fujisawa et al., 2011, 2012, 2013). More recently, gene editing of *RIN* has demonstrated that in the *RIN* CRISPR lines, the expression of many of these cell wall structure-related genes is somewhat suppressed. However, at later stages of ripening some genes encoding cell wall degrading enzymes, such as PG and TBG4, were expressed at higher levels in the *RIN* CRISPR lines than in wild type. Also, in the *RIN* CRISPR lines, the internal structure of the fruits visibly showed greater disruption and loss of integrity than wild type (Li et al., 2020b). These data indicate that RIN is involved in the control of genes involved in fruit softening and plays an important role in the fine control of the process.

In addition to RIN, NOR and CNR, a range of other TFs have been implicated in the regulation of tomato softening. A recent study showed that a new NAC-box transcription factor, NOR-like 1, influenced ripening related softening by directly targeting cell wall associated genes that degrade pectic polysaccharides such as PL and *POLYGALACTURONASE 2a* (PG2a) (Gao et al., 2018). The expression of a member of plant-specific GRAS gene family, designated as *SlFSR* (fruit shelf-life regulator) was highly ripening-related. Repression of *SlFSR* significantly reduced the expression of multiple cell wall modification-related genes including *PL*, *PG*, *TBG4*, *CEL2*, XYL1, *PECTIN ESTERASE* (PE), *MANNOSIDASE* (MAN1), *XYLOGLUCAN ENDOTRANSGLUCOSYLASE/HYDROLASE* (*XTH5*) and *EXP1*, and prolonged shelf life, but did not influence other aspects of fruit ripening. These observations reveal the potential role of SlFSR in targeted control of tomato fruit shelf life by regulating cell wall metabolism (Zhang et al., 2018).

Very recently two other tomato TFs have been implicated in softening in this fruit. Firstly, a *LATERAL ORGAN BOUNDRIES* (*LOB*) TF, *SlLOB1* has been shown by Shi to act as a transcriptional activator of a broad suite of cell wall–related genes and to control softening (Shi et al., 2021). Shi reported that repression of *SlLOB1* inhibits softening via reduced expression of multiple cell wall genes (Shi et al., 2021). In contrast, over expression of *SlLOB1* results in enhanced softening. In the second example, a master regulator involved in the light signalling pathway, SlHY5, has been shown to modulate ripening-related genes, including those involved in cell wall disassembly, at both transcriptional and translational level. ChIP-qPCR analysis indicated that promoters of cell wall related genes including *PG2A, PL1*, and *EXP1* as well as core regulators such as *RIN*, *CNR* and *FRUITFULL1* (FUL1) were direct targets of SlHY5. However, the interplay between SlHY5 and these regulators needs further investigation (Wang et al., 2021).

As mentioned earlier in this section more than 50 cell wall structure-related genes show altered expression during tomato fruit ripening. Linking these to texture changes in tomato has proved more of a challenge than was expected.

### Cell wall remodelling and fruit softening in tomato and other fleshy fruit

The major components of plant cell walls have been known for many years, but much remains to be discovered about the nanoscale assembly of these cell walls (Anderson & Kieber, 2020). The basic structural features involve three major classes of polysaccharides, cellulose, hemicellulose and pectin with a range of structural proteins also being present. The groups of cell wall polysaccharides are often discussed as independent entities, but there is strong evidence for close association of the components and covalent linkages among the different classes of molecules. The reader is referred to a recent review by Anderson and Kieber (2020) for a detailed summary of the most recent research.

*Polysaccharide domains and changes during ripening*- Alterations in pectin structure are consistently reported as being closely linked to softening (Posé et al., 2019). Pectic polysaccharides are composed principally of long chains of galacturonic acid (GAL) residues known as homogalacturonan (HG). These HG chains can be methyl esterified or deesterified and can be longer than 1000 nm in length. HG may be linked to regions where GAL residues are interspersed with rhamnose known as rhamnogalacturonan I (RGI) and there may also be side chains of galactose and arabinose residues. Recent models based on Atomic Force Microscopy (AFM) and modelling of molecular structures, suggest HG domains, unbranched or with a low number of HG branches, are associated with RG1 polymers of lower length (Paniagua et al., 2017).

Pectin depolymerisation is a common event in ripening fruits. In a recent review Posé et al. (2019) noted that ‘fruits with long pectin chains, [for example plum and apricot], share a consistent texture of relatively high firmness, in comparison with ripe strawberry, tomato or raspberry, with thinner and shorter pectin chains, that are characterised by a strong softening during ripening’. The length of the HG chains and numbers of rhamnose residues likely affects the gelling ability of the pectin domains (Pieczywek et al., 2020). Indeed, modelling of the conformation of pectin HG molecules showed that randomly dispersed HG chains had a tendency to aggregate into highly organised 3D structures. The final structure resembled a three-dimensional network created by tightly associated HG chains organised into thick fibres (Pieczywek et al., 2020). Long HG chains linked to RG1 cores may therefore have the potential to interact together to form HG nanofilaments and thick fibrils (Haas et al., 2020; Pieczywek et al., 2020). Ripening and softening in tomato and other fruits seems to involve solubilisation and degradation of this pectin network and pectin solubilisation can be directly related with the texture of ripe fruits (Posé et al., 2019).

There is evidence that pectic polysaccharides are covalently linked to other wall polysaccharide domains including RG-I –xylan interactions (Broxterman & Schols, 2018b). Studies of cell wall deposition indicate that a close association of pectin and cellulose may be a fundamental feature. During cell division wall components are sequentially delivered to the growing and developing cell plate. The sequence of deposition is positively charged extensin and negatively charged pectin that potentially interact electrostatically, followed by the deposition of callose, and finally the synthesis of cellulose (Phyo et al., 2017). Chemical analysis also supports a tight association of pectin and cellulose (Broxterman & Schols, 2018a), although the exact nature of this association is still a matter of debate.

In a similar way to the pectin domain, disassembly of the hemicellulose and cellulose domains in the wall also appear to be a common feature of ripening in tomato and other fruits, but the contribution of these events to texture changes is even less well understood (Posé et al., 2019). Studies in Arabidopsis are, however, providing important insights. Mechanical analysis and creeping tests of the xyloglucan-deficient mutants (*xxt1*/*xxt2*) in *Arabidopsis* (Park & Cosgrove, 2012a, 2012b) have challenged classic depictions of a “tethered network” model in which cellulose microfibrils are connected primarily by xyloglucan tethers and much of the cellulose surface is coated with xyloglucan in an extended conformation (Carpita & Gibeaut, 1993). Instead, a “biomechanical hotspot” model of primary cell wall structure has been proposed by Park and Cosgrove (2012a, 2012b), which suggests that there are limited xyloglucan-cellulose complexes between cellulose microfibrils and that these are likely to be the target of wall loosening proteins such as EXP (see next section). This idea has been tested further in *Arabidopsis* using nuclear polarisation enhanced Nuclear Magnetic Resonance (NMR) (Wang et al., 2013) and the data supports the conclusion that EXPs loosen cell walls by binding highly specific cellulose domains enriched in xyloglucan.

*Cell wall enzymes and other factors* - In both tomato and strawberry the expression of genes encoding enzymes with PL activity has been demonstrated to be important in softening of these fruits. In the absence of PL expression, tomato fruit softened more slowly and HG was retained at cell junctions in the pericarp (Uluisik et al., 2016; Yang et al., 2017; Wang et al., 2019). In strawberry, both PL and PG are important in normal softening and cell walls of transgenic fruits showed less depolymerisation of bound pectin as well as a lower degree of pectin solubilisation (Posé et al., 2019).

The action of PL on wall mechanical properties has been studied in onion cells. Here lateral mobility of cellulose microfibrils was greatly increased at the nm-scale after PL treatment. However, concomitant effects on wall loosening and tensile properties were absent (Zhang et al., 2019). These results indicate a role for HG in microfibril stability. In tomato, HG seems to accumulate in tricellular junctions and make up a fibrous material that is a major site of PL action in tomato (Uluisik et al., 2016). Removing any reinforcing HG zones at tricellular junction zones may help drive cell separation during fruit softening.

EXP1 has been shown to be important in tomato softening and there is an inhibitory effect on the process when *EXP1* is silenced (Brummell et al., 1999). The action of EXP1 in tomato likely reflects loosening of cellulose-xyloglucan interactions possibly at ‘biomechanical hotspots’ as described above. Pectin may also be important in the assembly of cellulose microfibrils and if so this could provide an explanation for the observation that silencing *EXP1* expression alters the extent of pectin depolymersation (Brummell et al., 1999). Apart from PL and EXP, it has proved difficult to identify other cell wall factors that have a proven role in tomato fruit softening. Wang compared tomato lines with PG, PL and a galactanase (TBG4) silenced by gene editing. Only the PL lines showed inhibited softening as determined by assessment of pericarp mechanical properties (Wang et al., 2019).

**Fig. 2:**
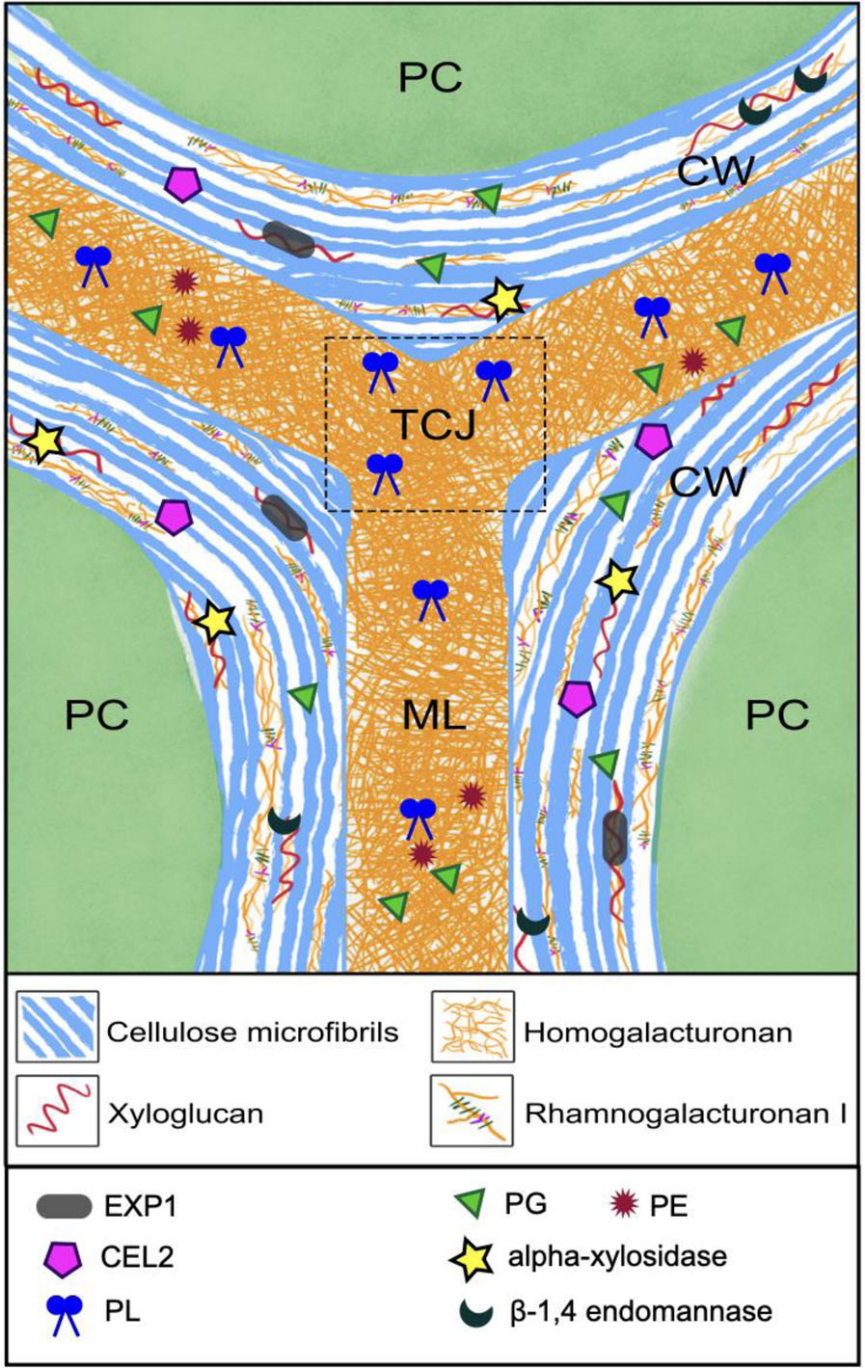
Tomato pericarp cell wall and some of the key enzymes involved in softening. Cellulose microfibrils are closely associated with pectin including homogalacturonan (HG) and rhamnogalacturonan I (RG-1I. HG chains can aggregate into highly organised structures and link to RG1 cores. The xyloglucans form “biomechanical hotspots” at limited locations in the CW, and tether cellulose microfibrils together. HG is present throughout the wall and especially in the middle lamella (ML) and the tricellular junctions (TCJ). Expansin, CEL2, alpha-xylosidase and β-1,4 endomannase are necessary for disrupting the associations between matrix polysaccharides and cellulose microfibrils. HG in TCJ and ML is disrupted by the combined activities of PL, PE and PG, allowing cell separation during tomato fruit softening. Based on evidence from tomato and other plant species including work reported by Paniagua et al. (2017), Pieczywek et al. (2020), Uluisik et al. (2016), Wang et al. (2019) and Shi et al. (2021)

New insights into the mechanisms involved in cell wall disassembly are now coming from studies of ripening-related TFs. Shi investigated the genes impacted by suppressing or upregulating the expression of the TF *SlLOB1* (Shi et al., 2021). Transcript and protein levels of *EXP1* are strongly suppressed in *SILOB1* RNA interference lines and induced in *SlLOB1-*overexpressing fruits. Other cell wall-related genes that show related patterns of expression in the *SlLOB1* lines included *CEL2* which encodes an endo-β1,4-glucanase and genes encoding an alpha-xylosidase and a β-1,4 endomannase. As already mentioned, EXPs may enhance the ability of cellulase to act on cellulose microfibrils (Zhang et al., 2021) and EXPs and CEL2, alpha-xylosidase and β-1,4 endomannase likely promote disruption of non-covalent and covalent links between matrix polysaccharides and microfibrils (Shi et al., 2021). It seems possible that in tomato, PL and the cell wall factors influenced by SlLOB1, may account for many of the components controlling normal softening. They act in concert to disrupt interactions between cellulose and pectin and also breakdown pectin aggregates and fibrils that are important in cell-to-cell adhesion (Fig. 2). These events result in alterations in cell wall mechanical properties and cell separation perceived as texture changes by the consumer. To develop a more comprehensive mechanistic model of softening in tomato and other fruits a better understanding of the nanoscale assembly of plant cell walls is now required.

## Data Availability

Not Applicable to this article as no datasets were generated or analysed during the current study.

## Abbreviations

SlCYP707A2: ABA 8′-hydroxylase
ABA: abscisic acid
SlALKBH2: Arabidopsis m6A RNA demethylase
AFM: Atomic Force Microscopy
CpARF2-CpEIL1: Auxin Response Factors-Ethylene Insensitive3-Like
CEL1: Cellulase 1
CEL2: Cellulase 2
SlNCED1: 9-cis-epoxycarotenoid dioxygenase
CNR: Colourless non-ripening
DML: DEMETER-like DNA demethylase
LeMAN4: Endo-(1,4)-β-mannanase 4
EXP: Expansin
LeEXP1: tomato α - Expansin 1
TBG4: tomato galactanase / β-Galactosidase 4
GAL: Galacturonic acid
GA: Gibberellins
HG: Homogalacturonan
LOB: LATERAL ORGAN BOUNDRIES
MAN1: mannosidase
ncRNAs: non-coding RNAs
NOR: non-ripening
PRC1: PcG protein SlMSI1 Polycomb Repressive Complex
PL: pectate lyase
PE: PECTIN ESTERASE
SlFSR: Fruit shelf-life regulator
PcG: Polycomb-group proteins
PG: polygalacturonase
PG2a: POLYGALACTURONASE 2a
SlPYL9: pyrobactin resistance-like
FIS1: FIRM SKIN 1
RGI: Rhamnogalacturonan I
FaRGlyase1: rhamnogalacturonate lyase
RIN: RIPENING INHIBITOR gene
rin: Ripening inhibitor mutant
SlHY5: ELONGATED HYPOCOTYL 5
FUL1: FRUITFULL1
TF: Transcription Factor
WGD: whole genome-duplication
XYL1: xylan 1,4-beta-xylosidase1
XTH5: xyloglucan endotransglucosylase/hydrolase 5
MYB5: v-myb avian myeloblastosis viral oncogene homologue
